# Community transmission of multidrug-resistant tuberculosis is associated with activity space overlap in Lima, Peru

**DOI:** 10.1186/s12879-021-05953-8

**Published:** 2021-03-18

**Authors:** David P. Bui, Shruthi S. Chandran, Eyal Oren, Heidi E. Brown, Robin B. Harris, Gwenan M. Knight, Louis Grandjean

**Affiliations:** 1 Department of Epidemiology and Biostatistics, The University of Arizona, Mel and Enid Zuckerman College of Public Health, 1295 N Martin Ave., Tucson, AZ 85724 USA; 2 The London School of Hygiene and Tropical Medicine, Keppel Street, London, UK; 3 San Diego State University, School of Public Health, 5500 Campanile Drive, San Diego, California 92182 USA; 4 Universidad Peruana Cayetano Heredia, Lima, Peru; 5 Institute of Child Health, University College London, 30 Guilford Street, London, UK

**Keywords:** Tuberculosis, Community transmission, GPS, Home range, Activity space

## Abstract

**Background:**

Transmission of multidrug-resistant tuberculosis (MDRTB) requires spatial proximity between infectious cases and susceptible persons. We assess activity space overlap among MDRTB cases and community controls to identify potential areas of transmission.

**Methods:**

We enrolled 35 MDRTB cases and 64 TB-free community controls in Lima, Peru. Cases were whole genome sequenced and strain clustering was used as a proxy for transmission. GPS data were gathered from participants over seven days. Kernel density estimation methods were used to construct activity spaces from GPS locations and the utilization distribution overlap index (UDOI) was used to quantify activity space overlap.

**Results:**

Activity spaces of controls (median = 35.6 km^2^, IQR = 25.1–54) were larger than cases (median = 21.3 km^2^, IQR = 17.9–48.6) (*P* = 0.02). Activity space overlap was greatest among genetically clustered cases (mean UDOI = 0.63, sd = 0.67) and lowest between cases and controls (mean UDOI = 0.13, sd = 0.28). UDOI was positively associated with genetic similarity of MDRTB strains between case pairs (*P* < 0.001). The odds of two cases being genetically clustered increased by 22% per 0.10 increase in UDOI (OR = 1.22, CI = 1.09–1.36, P < 0.001).

**Conclusions:**

Activity space overlap is associated with MDRTB clustering. MDRTB transmission may be occurring in small, overlapping activity spaces in community settings. GPS studies may be useful in identifying new areas of MDRTB transmission.

**Supplementary Information:**

The online version contains supplementary material available at 10.1186/s12879-021-05953-8.

## Introduction

Tuberculosis (TB) is commonly transmitted outside of the home in community-based settings during social contact between infectious cases and susceptible community members [1, 2]. Prior studies have used questionnaires to identify epidemiologic links between unrelated TB cases and have found spatial links between cases in community settings [3–6]. Identifying spatial areas of transmission is important for contact tracing and infection control, as epidemiologic links between TB cases are often unclear [7, 8]. Moreover, targeting infection control in geographic hotspots of TB transmission may reduce overall levels of community transmission [9].

Activity spaces are used in epidemiologic studies to represent geographic spaces wherein people spend their time during regular daily activities [10–12]. Past studies of human activity spaces have relied predominantly on place-tracing questionnaires to delineate activity spaces [3–5, 13]. More recent approaches now leverage global positioning systems (GPS) [10, 14, 15]. The widespread availability of GPS technology has facilitated fine scale study of human movement patterns and are less prone to recall and measurement errors common in retrospective place-tracing interviews [16–19].

The goal of this study was to compare the activity spaces of multidrug resistant tuberculosis (MDRTB) cases and healthy (TB-free) community controls, identify areas of activity space overlap among clustered cases to identify areas of potential transmission, and to quantify the association between activity space overlap and genetic clustering of MDR *Mycobacterium tuberculosis* (Mtb) strains.

## Methods

### Study design and setting

Study participants were recruited from the areas of Callao and Lima Sur located to the north and south of Lima, Peru, respectively. These two regions report the greatest proportion of incident MDRTB cases in Peru [20]. Callao has an area of 147 km^2^ with nearly all one million residents living in urban areas. Lima Sur encompasses 11 districts with a total area of 852 km^2^ and 1.5 million residents.

Between February 2016 and May 2017, patients were recruited from a completed parent study that enrolled a household-based cohort of MDRTB patients between 2010 and 2013 [21]. Sputum samples from cases were taken at diagnosis and processed on liquid microscopic observation drug susceptibility assays (MODS) and solid Ogawa media. Aliquots of positive sputum samples were reserved for DNA extraction and genotyping via whole genome sequencing [22]. The single nucleotide polymorphism (SNP) calling analysis was performed on an Illumina HiSeq2000 with paired-end reads of length of 100 bp [23]. A pairwise matrix of MDRTB cases and the number of single nucleotide polymorphism (SNP) differences in their Mtb strains was assembled; cases with Mtb strains within ≤5 SNP differences were considered genetically clustered [24]. This threshold was our working definition for MDRTB transmission. Exclusion criteria included genetically clustered pairs from the same household. Where a case was genetically clustered to multiple cases from the same household, only one member of that household was enrolled.

We enrolled community controls, who verbally confirmed that they had never received TB treatment or diagnosis, as a comparison sample. Controls included community health workers and nurses that worked at community health posts serving case neighborhoods. Additional controls were referred by community health workers and sourced from churches, restaurants, communal kitchens and education centers located in case neighborhoods. Controls were frequency matched to cases on age (± 5 years), sex (male/female) and study region (Callao/Lima Sur) to ensure comparability across these variables.

Informed consent was obtained from participants prior to data collection. The study protocol, consent forms and data collection instruments were reviewed and approved by the Institutional Committee of Ethics for Humans at La Universidad Peruana Cayetano Heredia.

### Data sources and measurements

Questionnaires were used to collect demographic information from participants during face-to-face interviews. We used Qstarz BT-Q1000XT (Qstarz International, Taipei, Taiwan) GPS loggers to gather data on participant’s movements over seven days of observation. The units were configured to log participant’s locations (i.e., geocoordinates) every minute. Consenting participants were given a GPS logger and instructed to keep the logger powered on, carried with them at all times, and recharged nightly. Study nurses called participants every other day during the 7-day data collection period to remind them to carry GPS loggers.

### Constructing activity spaces

Spatial ecologists have developed a suite of methods to study movement patterns of wildlife using GPS technology to delineate areas of regular space use called ‘home ranges’ and are analogous to activity spaces in human research [25]. These methods account for non-uniform space use by representing home ranges (i.e., activity spaces) as spatial probability density functions of space use called utilization distributions (UD) [26]. Instead of assuming space-use is uniform across an activity space, UDs highlight areas of concentrated activity with probability contours and are better representations of space use. In this study, we use home ranges to represent participant activity spaces.

Kernel density estimation (KDE) was used to construct participant activity spaces [25, 27]. We used iterative visualizations of GPS kernel densities generated with a Gaussian kernel and bandwidths between 100 m and 1200 m to identify UDs that provided adequate smoothing of raw GPS locations while highlighting distinct “peaks” of areas where locations were concentrated. The final chosen bandwidth was 950 m.

Home range (i.e., activity space) sizes were estimated at 50, 95 and 99% contours of each participant’s UD. These percentages correspond to the smallest home range area encompassing 50, 95 and 99% of a participant’s GPS locations. The 95% contour is the standard used in home range studies, while the 50% contour is considered the “core area” of activity [28, 29]. The 99% contour is the most inclusive contour, containing areas of sparse activity.

### Measuring spatial overlap

After estimating the UDs to represent each participant’s activity space, we calculated the utilization distribution overlap index (UDOI) to quantify the amount of activity space spatial overlap between participants [26]. The UDOI is estimated as the cumulative sum of the cell-by-cell product of two participant UDs multiplied by the intersecting area (i.e., product of two UDs) [26, 28]. The UDOI of two participants is high when their GPS locations are concentrated within the same space [26]. The UDOI ranges from 0 (no spatial overlap) to 1 (complete spatial overlap) and can take on values > 1 if the UDs are non-uniformly distributed and have a high degree of overlap. We estimated the UDOI’s at each home range contour level (50, 95, and 99%) to examine the magnitude of association between MDRTB transmission and activity space overlap. We created UDs for each participant and estimated the UDOIs for all pairs of participants using the ‘adehabitatHR’ package [30] in R (version 3.6.1, The R Foundation).

### Statistical analyses

T-tests, Wilcoxon rank sum tests (when appropriate), and chi-squared tests were used to compare cases and controls by demographics, home range size and mean UDOI.

We compared the mean pairwise UDOI of cases and controls to determine the degree of spatial overlap within and between groups. The mean UDOI of case dyads (i.e., case-case pairs), control dyads (i.e., control-control pairs), and case and control dyads (i.e., case-control pairs) were evaluated. Bonferroni adjusted *P*-values were reported to account for multiple comparisons.

We used logistic regression to estimate the odds of being genetically clustered as a function of spatial overlap (among cases). Linear regression was used to assess the relationship between the UDOI of case pairs and degree of genetic strain similarity (i.e., SNP differences). SNP difference values were log (base 10) transformed and logit transformed UDOI values were used to meet normality assumptions for modelling.

## Results

### Comparing cases and controls

A total of 99 participants were enrolled, including 35 MDRTB cases (35%) and 64 healthy community controls (65%). Only 35 participants of the original study could be contacted and consented, as the majority were no longer contactable, had moved house or had died. Sixteen (46%) cases were genetically clustered with matching *Mycobacterium tuberculosis* (Mtb) strains (within ≤5 SNPs). Frequency matching between cases and controls was achieved by region, age and sex (Table 1). The median age, gender and regional distribution of participants in both case and control groups were comparable, though not found to be statistically significant. Participants in both groups had similar levels of employment, but participants in the case group generally had a lower level of educational attainment compared to controls (31.4% vs 6.3% had primary education or less). The case group had a lower monthly income compared to the control group (77.1% vs 20.3% earned less than 1000 PEN/month). The differences in education and income were statistically supported. The sublineages of Mtb strains included in our study cohort were compared with those from the parent study population (Table S1). There was a similar distribution of sublineages represented between the two studies.

**Table 1:** Summary of study participants and comparison of multidrug resistant tuberculosis (MDRTB) cases and TB-free community controls enrolled in this study

	Cases (***N*** = 35)	Controls (***N*** = 64)	Total (***N*** = 99)	***P***-Value
**Age (years), mean (sd)**	34.9 (15.0)	35.5 (14.4)	35.3 (14.5)	0.86
**Female, n (%)**	16 (45.7)	25 (39.1)	41 (41.4)	0.52
**Study Region, n (%)**				0.68
Callao	16 (45.7)	32 (50)	48 (48.5)	
Lima Sur	19 (54.3)	32 (50)	51 (51.5)	
**Employed, n (%)**	24 (68.6)	47 (73.4)	71 (71.7)	0.52
**Primary Education or Less, n (%)**	11 (31.4)	4 (6.3)	15 (15.2)	0.001
Income < 1000 PEN/month*, n (%)	27 (77.1)	13 (20.3)	40 (40.4)	< 0.001
**GPS Data, median (IQR)**				
GPS relocations (thousands)	7.8 (4.7–9.5)	8.4 (6.6–9.9)	8.3 (5.6–9.8)	0.13
Hours of Tracking (hundreds)	1.3 (0.8–1.6)	1.4 (1.1–1.6)	1.4 (0.9–1.6)	0.13
**Home Range Size (km2), median (IQR)**				
50% Home Range Contour	4.3 (4.0–7.0)	5.3 (4.4–7)	5.0 (4.2–7.0)	0.02
95% Home Range Contour	21.3 (17.9–48.6)	35.6 (25.1–54)	32.3 (19.8–53.5)	0.02
99% Home Range Contour	49.4 (28–94.3)	72.5 (45.7–127.8)	64.7 (35–113.3)	0.05

* PEN = Peruvian Nuevo Sol

A median of 8253 GPS locations (IQR 5634-9828) were collected per participant, representing about 138 h of data (IQR 94–164) per participant. A median of 7756 GPS locations (IQR 4813-9493), equivalent to 129 h (IQR 80–158), were collected for cases and a median of 8364 GPS locations (IQR 6766-9887), equivalent to 139 h (IQR 113–165), were collected for controls. There were, however, no statistically significant differences in the amount of GPS locations collected between cases and controls.

### Home ranges of cases and controls

**Fig. 1:**
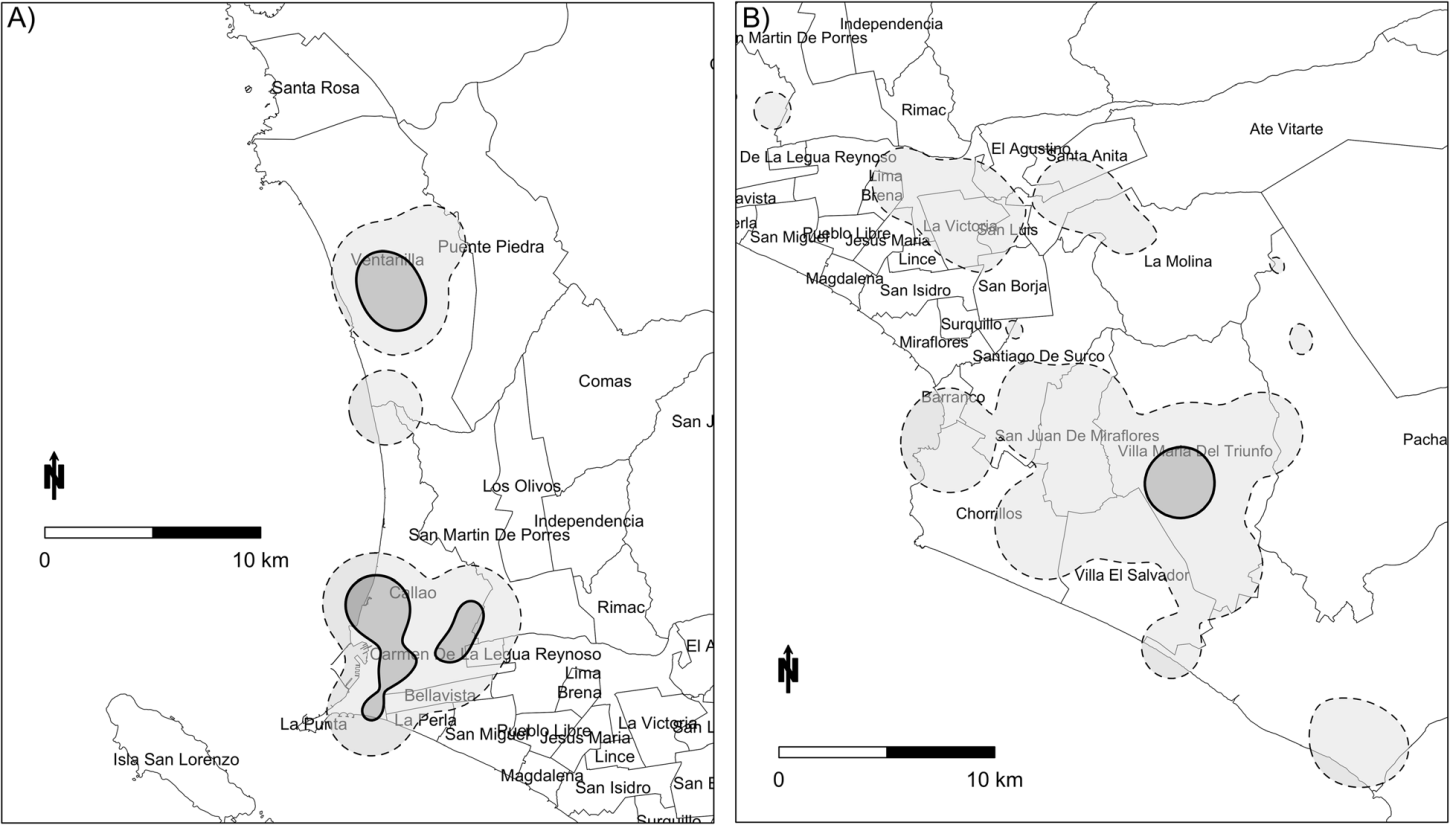
District maps of Lima, Peru illustrating home ranges in Callao and Lima Sur. **a** home ranges of all MDRTB cases in Callao and **b**) home ranges of all MDRTB cases in Lima Sur. The 50% home range contours are drawn with a thick solid border and 95% home range contours are drawn with a dashed line

The aggregated 50 and 95% home ranges of all cases in Callao and Lima Sur are shown in Fig. 1.

In Callao, the 50% home range of cases were located on either side of Jorge Chávez International Airport in high traffic corridors and densely populated neighborhoods; another 50% core was located in a densely commercial area of Ventanilla. In Lima Sur, there was one 50% home range core located in the district of Villa Maria Del Triunfo. Case home ranges were significantly smaller than control home ranges at all contour levels (Table 1).

### Spatial overlap between cases and controls

**Fig. 2:**
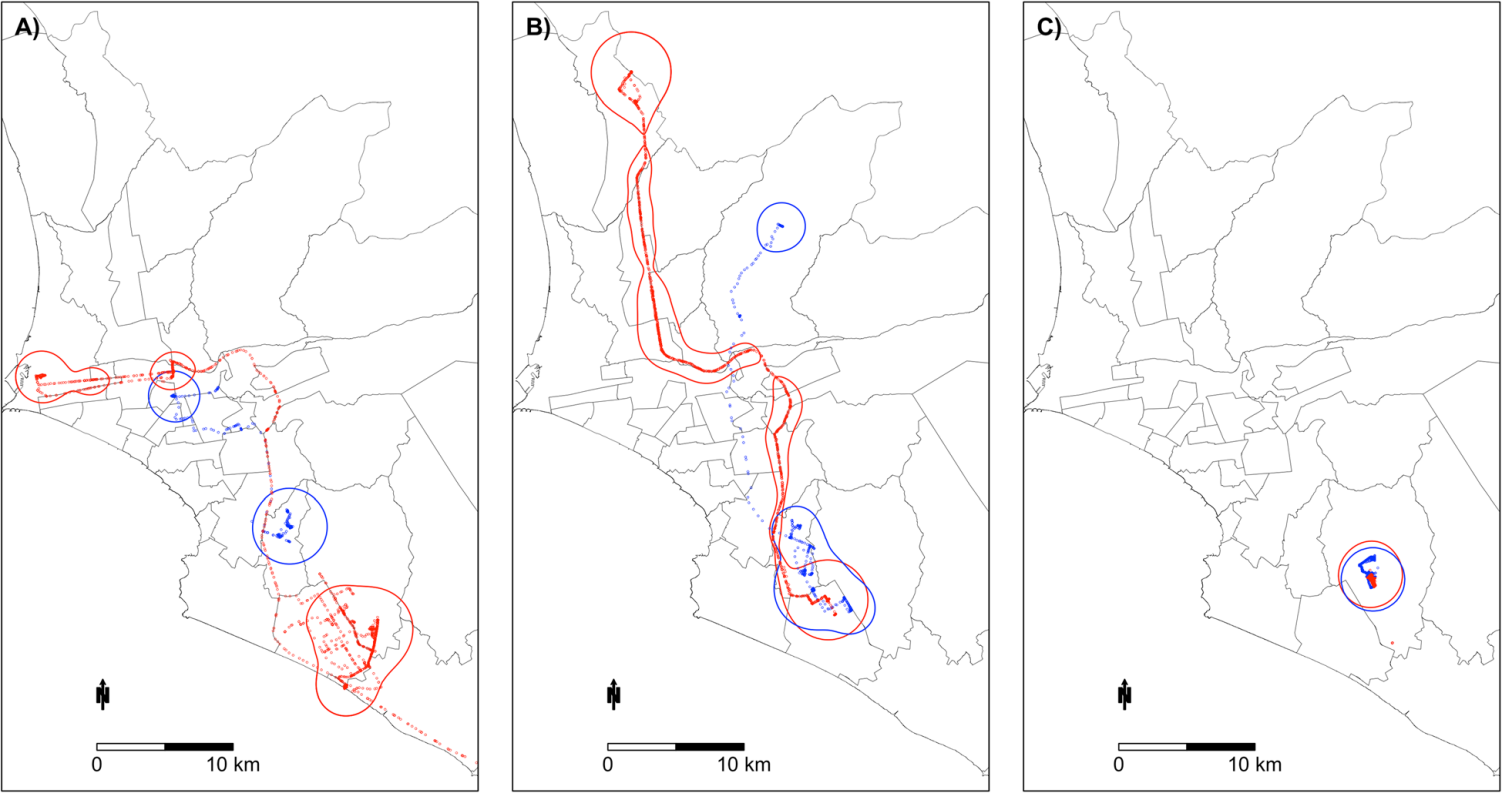
Three examples of home ranges with differing 95% home range overlaps. Low (Panel **a**), moderate (Panel **b**) and high (Panel **c**) 95% home range overlaps measured by their utilization distribution overlap index (UDOI). The small circles are GPS relocation trails and the larger outlines are the 95% home ranges of each case. The blue and red coloring are used to differentiate the two cases. Map outline is of districts in Lima. Panel **a** shows two (non-clustered) cases with very low overlap (UDOI< 0.01); Panel **b** shows two (non-clustered) cases with moderate overlap (UDOI = 0.63) and Panel **c** shows two (clustered) cases with very high overlap (UDOI> 1.0)

Table 2 summarizes the mean UDOI by dyad type (i.e., pairs of participant types) at the 95% home range contour. Mean UDOI was greatest among clustered case dyads (mean = 0.29, sd = 0.55) in all regions whereas mean UDOI was lowest among non-clustered cases (mean = 0.05, sd = 0.20) (*P* < 0.01). Mean UDOI was higher among controls (mean = 0.14, sd = 0.36) than among cases (mean = 0.06, sd = 0.22) (*P* < 0.001). Figure 2 provides three illustrative examples of low, medium and high UDOI values for three dyads of cases. There was a high proportion of non-overlapping dyads (between 35 to 47% of possible dyads that did not have overlapping activity spaces). As a sensitivity analysis, we analyzed the mean UDOI of dyad types with only overlapping dyads and found that mean UDOI increased across all dyad types, but there was no change in inferences (Table 2).

**Table 2:** Comparison of mean 95% utilization distribution overlap index (UDOI) values by dyad type and study regions (Callao and Lima Sur)

Dyad Type	N Dyads	No Overlap, n (%)	Overlap, n (%)	All Dyads 95% UDOI, mean (sd)	Overlapping Dyads Only 95% UDOI, mean (sd)
**Both Regions**					
Both Cases	595	385 (64.7)	210 (35.3)	0.06 (0.22)	0.17 (0.34)
Both Controls	2016	1089 (54.0)	927 (46.0)	0.14 (0.36)	0.31 (0.47)
Case-Control	2240	1311 (58.5)	929 (41.5)	0.06 (0.19)	0.13 (0.28)
Clustered	15	8 (53.3)	7 (46.7)	0.29 (0.55)	0.63 (0.67)
Not Clustered	580	377 (65.0)	203 (35.0)	0.05 (0.20)	0.15 (0.31)
**Callao Only**					
Both Cases	120	68 (56.7)	52 (43.33)	0.12 (0.33)	0.29 (0.45)
Both Controls	496	126 (25.4)	370 (74.6)	0.29 (0.43)	0.39 (0.46)
Case-Control	512	232 (45.3)	280 (54.69)	0.09 (0.23)	0.16 (0.29)
Clustered	4	2 (50.0)	2 (50)	0.63 (0.81)	1.26 (0.63)
Not Clustered	116	66 (56.9)	50 (43.1)	0.11 (0.29)	0.25 (0.40)
**Lima Sur Only**					
Both Cases	171	36 (21.1)	135 (78.9)	0.12 (0.27)	0.15 (0.30)
Both Controls	496	34 (6.9)	462 (93.2)	0.3 (0.49)	0.32 (0.50)
Case-Control	608	79 (13.0)	529 (87.0)	0.13 (0.28)	0.14 (0.30)
Clustered	7	2 (28.6)	5 (71.4)	0.27 (0.48)	0.38 (0.55)
Not Clustered	164	34 (20.7)	130 (79.3)	0.11 (0.26)	0.14 (0.29)

### Association between spatial overlap and genetic clustering

There was a statistically significant positive association between the UDOI of cases and genetic clustering (i.e. being part of a recent transmission chain). At the 95% contour, the odds of two cases being clustered increased by 22% per 0.10 increase in their shared UDOI (OR = 1.22, CI = 1.09–1.36, P < 0.001). The effect was larger at the 50% contour where the odds of two cases being clustered was increased by four-fold for every 0.10 increase in UDOI (OR = 4.25, CI = 1.85–9.73, *P* = 0.001) (Table 3).

**Table 3:** Association between genetic similarity of *Mycobacterium* strains of two case pairs (measured in number of different single nucleotide polymorphisms (SNP) and their utilization distribution overlap index (UDOI)

**Linear regression of Log (SNP difference) on logit (UDOI).**	**Coef.**	**95%CI**		**P**
50% UDOI	−0.87	−1.35	−0.40	< 0.001
95% UDOI	−2.59	−3.80	−1.37	< 0.001
99% UDOI	−3.57	−5.00	−2.14	< 0.001
**Odds of being in a clustered dyad vs. a non-clustered dyad by UDOI.**	**OR**	**95%CI**		**P**
50% UDOI	4.25	1.85	9.73	0.001
95% UDOI	1.22	1.09	1.36	< 0.001
99% UDOI	1.10	1.04	1.16	0.001

**Fig. 3:**
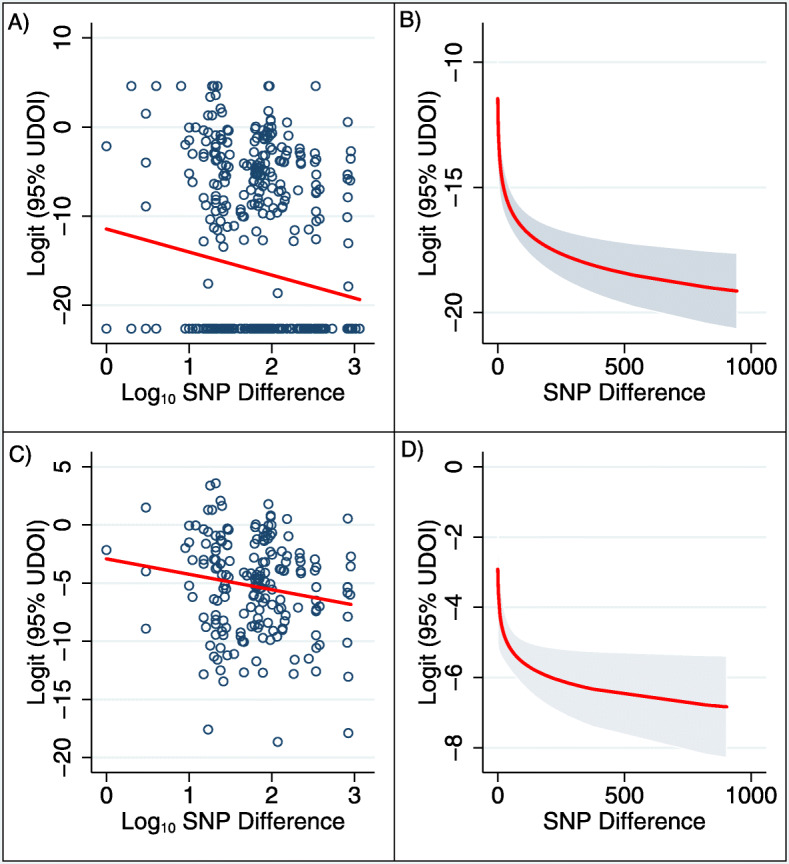
Two-way scatter plots and linear regression predictions of 95% UDOI and SNP difference. **a** Two-way scatter plot of 95% UDOI and SNP difference between case pairs, and **b**) linear regression prediction of 95% UDOI and SNP difference with confidence interval. There is a statistically significant negative association between UDOI and SNP difference (Coef = − 2.59, *P* < 0.001). Note UDOI is logit transformed to reduce variable skew for regression modelling. Panel **c** and **d** exclude case pairs with no overlap

There was a statistically significant association between spatial overlap and Mtb genetic similarity (i.e., SNP differences) among cases (Fig. 3a and b). At the 95% home range contour, for every log increase in the number of SNP difference, there was a 2.6-unit reduction in the logit transformed UDOI (coef = − 2.6, CI = -3.8–1.4, P < 0.001) or approximately 0.0025 reduction in UDOI (Table 3). In other words, as genetic similarity of two Mtb strains increased, their level of home range overlap increased.

Since there was a large proportion of non-overlapping dyads that might have influenced the negative association between SNP difference and UDOI, we conducted a sensitivity analysis excluding non-overlapping dyads and found the association between Mtb genetic dissimilarity and UDOI of two cases attenuated but was still statistically significant (Fig. 3c and d). During post-hoc review, we found one clustered dyad of related family members, resulting in a very high UDOI that may be considered an outlier. After excluding this dyad from analysis, the mean UDOI of clustered cases was reduced, but remained greater than all other dyad pairs (Table S2). The association between the genetic similarity of two Mtb strains and UDOI was slightly attenuated, but remained statistically significant at all home range levels (Table S3).

## Discussion

In this study, we compared the activity spaces of MDRTB cases to healthy community controls. We found that cases had significantly smaller activity spaces than controls, that the activity space overlap was greatest among genetically clustered cases, and that there was a statistically significant association between activity space overlap and Mtb strain genetic similarity. These findings suggest that MDRTB contact, exposure and transmission may be occurring among cases in relatively small, overlapping activity spaces in community settings.

The demonstration of high overlap amongst genetically clustered cases and lower overlap between cases and controls in the dyad analysis suggests that spatial segregation between the groups may be occurring, as found in a previous study [31]. Spatial segregation of MDRTB cases may support the use of spatially targeted screening interventions to improve local control and indirectly reduce MDRTB prevalence [32]. The high spatial overlap among cases may explain why spatial clustering of MDRTB genotypes has been previously found in localized hotspots in Lima [2]. The association between Mtb genetic similarity and spatial overlap was observed at all home range contours but was particularly pronounced at the smaller 50% home range contour (median of 5km^2^), suggesting that transmission of MDRTB may be occurring at very local levels near case residences. Yang et al. found that genetic similarity of Mtb strains of MDRTB case pairs in China increased as their residential proximity increased [33]. A phenomenon of MDRTB spillover from a prison in Lima to the surrounding population was demonstrated by Warren et al. and proposes a mechanism whereby local transmission may be occurring in the community [34]. Moreover, as clustered cases tended to have substantially smaller activity spaces than non-clustered cases, this suggests that movement and spatial overlap in small geographic hotspots may be driving MDRTB transmission in this population. Focusing infection control in those areas of high overlap may reduce MDRTB transmission and result in community-wide benefits as suggested by Dowdy et al. [9]. It is important to recognize, however, that drivers of transmission hotspots are likely to vary between rural and urban environments. Nelson et al. demonstrated that transmission of extensively drug resistant TB (XDRTB) was likely occurring far away from cases’ residences in rural South Africa. The authors suggest that cyclical rural-to-urban migration for work was an important determinant of transmission (Nelson et al. 2018). Shift of XDRTB transmission towards workplaces in urban centers in Durban was also highlighted by Peterson et al. [13].

To date, studies investigating transmission sites for TB have relied on questionnaires about frequented locations [6], which generally underestimate spatial mobility and are subject to information bias [16, 18]. This study uses GPS tracking to obtain objective spatial information on participants and does not rely on participants’ recall. Activity spaces of individuals, calculated using GPS logging data, were shown to be larger than those derived from geotagging venues reported in questionnaires [35]. Moreover, place-tracing questionnaires often focus only on community venues and do not take into account routes travelled between them [35]. This suggests that GPS methods are superior in acquiring a greater amount of spatial information. This methodology is easily reproducible and demonstrates the utility of GPS tracking in combination with whole genome sequencing to identify potential transmission sites.

There are several limitations to our study that should be noted. Firstly, it is likely that selection bias was introduced through our non-random selection of controls. As this was an exploratory study, our sample of controls were convenience-based and were often health workers or their family and friends. As a result, they generally had a higher level of education and income and were not necessarily representative of the general population. Moreover, given the higher socioeconomic status (SES) of controls, it is likely that controls did not live in the same neighborhoods nor frequent the same shops and venues that cases did, which may have resulted in the observed low UDOI between case and control dyads (but the relatively higher UDOI among control-dyads). We attempted to address this source of bias through matching controls by age, sex and study region, but this was not sufficient to control for confounding caused by SES. Our healthy controls were also recruited on the basis that they had never been treated or diagnosed with previous TB. However, this was not confirmed by medical records and may represent another potential source of bias. Our analysis of genetic clusters relied on a SNP difference threshold to determine which cases were genetically clustered, representative of recent transmission [24]. While a small SNP difference between two *M. tuberculosis* strains is generally regarded as evidence of transmission, the appropriate clustering threshold depends on the environment and setting [36]. While GPS monitoring is considered to be more precise than structured interviews at identifying activity locations, GPS locations alone do not provide context for locations (i.e., types of places or reasons for visiting areas) that interviews could elicit; combining the two forms of data collection is preferable [15, 16]. On average, study participants provided slightly less than seven days of GPS location data, so these movement patterns might not be representative of typical activity. However, prior studies have found that human movement patterns tend to be regular and stable, particularly in urban settings where routines are structured and people tend to spend significant amounts of time in few, regularly visited locations [18, 25, 37].

The small sample size was another limitation to the study. This was mainly due to the large numbers of potential participants who had moved or had unfortunately died. The findings of this study can therefore only be regarded as exploratory in nature.

A larger prospective study with a bigger sample size would be useful to confirm our findings and determine whether the differences between cases and controls, in terms of activity space size and overlap likelihood at all activity space contour sizes, are statistically meaningful. Potential sources of bias could be reduced through the recruitment of confirmed TB-free controls from cases’ neighbourhoods. A detailed investigation into community venues within overlapping activity spaces of genetically-clustered participants is essential to isolate specific areas where transmission is occurring. Additionally, follow-up questionnaires conducted alongside GPS telemetry would be useful to characterise the context of visits to community venues. Data collection could be made simpler and more effective by using alternative sources of GPS data, such as Google maps location history on participants’ smartphones. Data on the movement patterns of TB patients during the period of transmission itself may provide greater insight into specific locations where transmission may have occurred.

## Conclusion

In Lima, Peru, activity space overlap is associated with genetic clustering of MDRTB cases and case activity spaces are relatively small. Case finding activities should focus on areas within 5 km of case residences which is the core area of movement for cases and where community transmission may be most likely.

## Supplementary Information

**Additional file 1: Table S1.** Distribution of sublineages in study and parent study. Fisher-exact *P*-value = 0.83.

**Additional file 2: Table S2.** Comparison of mean 95% UDOI by dyad type, excluding clustered cases from the same household.

**Additional file 3: Table S3.** Association between genetic similarity of *Mycobacterium tuberculosis* strains of two case pairs (measured in number of different single nucleotide polymorphisms (SNP)) and their utilization distribution overlap index (UDOI), excluding clustered cases that are household members.

## Data Availability

The datasets used and/or analysed during the current study are available from the corresponding author on reasonable request.

## Abbreviations

GPS: Global positioning systems
KDE: Kernel density estimation
MDR: Multidrug resistant
MODS: Microscopic observation drug susceptibility assays
Mtb: *Mycobacterium tuberculosis*
SNP: Single nucleotide polymorphism
TB: Tuberculosis
UD: Utilization distribution
UDOI: Utilization distribution overlap index
XDR: Extensively drug resistant

## References

[1] Yates TA, Khan PY, Knight GM, Taylor JG, McHugh TD, Lipman M (2016). The transmission of Mycobacterium tuberculosis in high burden settings. Lancet Infect Dis.

[2] Zelner JL, Murray MB, Becerra MC, Galea J, Lecca L, Calderon R (2015). Identifying hotspots of multidrug-resistant tuberculosis transmission using spatial and molecular genetic data. J Infect Dis.

[3] Chamie G, Wandera B, Marquez C, Kato-Maeda M, Kamya MR, Havlir DV (2015). Identifying locations of recent TB transmission in rural Uganda: a multidisciplinary approach. Trop Med Int Heal.

[4] Chamie G, Kato-Maeda M, Emperador DM, Wandera B, Mugagga O, Crandall J (2018). Spatial overlap links seemingly unconnected genotype-matched TB cases in rural Uganda. PLoS One.

[5] Izumi K, Ohkado A, Uchimura K, Murase Y, Tatsumi Y, Kayebeta A (2015). Detection of tuberculosis infection hotspots using activity spaces based spatial approach in an urban Tokyo, from 2003 to 2011. PLoS One.

[6] Shaweno D, Karmakar M, Alene KA, Ragonnet R, Clements AC, Trauer JM (2018). Methods used in the spatial analysis of tuberculosis epidemiology: a systematic review. BMC Med.

[7] Gardy JL, Johnston JC, Sui SJH, Cook VJ, Shah L, Brodkin E (2011). Whole-genome sequencing and social-network analysis of a tuberculosis outbreak. N Engl J Med.

[8] Klovdahl AS, Graviss EA, Yaganehdoost A, Ross MW, Wanger A, Adams GJ (2001). Networks and tuberculosis: an undetected community outbreak involving public places. Soc Sci Med.

[9] Dowdy DW, Golub JE, Chaisson RE, Saraceni V (2012). Heterogeneity in tuberculosis transmission and the role of geographic hotspots in propagating epidemics. Proc Natl Acad Sci U S A.

[10] Hirsch JA, Winters M, Clarke P, McKay H (2014). Generating GPS activity spaces that shed light upon the mobility habits of older adults: a descriptive analysis. Int J Health Geogr.

[11] Sherman JE, Spencer J, Preisser JS, Gesler WM, Arcury TA (2005). A suite of methods for representing activity space in a healthcare accessibility study. Int J Health Geogr.

[12] Worrell MC, Kramer M, Yamin A, Ray SM, Goswami ND. Use of activity space in a tuberculosis outbreak: bringing homeless persons into spatial analyses. Open Forum Infect Dis. 2017;4. 10.1093/ofid/ofw280. 10.1093/ofid/ofw280 PMC5414060 28480272

[13] Peterson ML, Gandhi NR, Clennon J, Nelson KN, Morris N, Ismail N (2019). Extensively drug-resistant tuberculosis “hotspots” and sociodemographic associations in Durban, South Africa. Int J Tuberc Lung Dis.

[14] Lee NC, Voss C, Frazer AD, Hirsch JA, McKay HA, Winters M (2016). Does activity space size influence physical activity levels of adolescents?—a GPS study of an urban environment. Prev Med Reports.

[15] Shareck M, Kestens Y, Gauvin L (2013). Examining the spatial congruence between data obtained with a novel activity location questionnaire, continuous GPS tracking, and prompted recall surveys. Int J Health Geogr.

[16] Paz-Soldan VA, Reiner RC, Morrison AC, Stoddard ST, Kitron U, Scott TW (2014). Strengths and weaknesses of global positioning system (GPS) data-loggers and semi-structured interviews for capturing fine-scale human mobility: findings from Iquitos. Peru PLoS Negl Trop Dis.

[17] Thierry B, Chaix B, Kestens Y (2013). Detecting activity locations from raw GPS data: a novel kernel-based algorithm. Int J Health Geogr.

[18] Vazquez-Prokopec GM, Bisanzio D, Stoddard ST, Paz-Soldan V, Morrison AC, Elder JP (2013). Using GPS technology to quantify human mobility, Dynamic Contacts and Infectious Disease Dynamics in a Resource-Poor Urban Environment. PLoS One.

[19] Vazquez-Prokopec GM, Stoddard ST, Paz-Soldan V, Morrison AC, Elder JP, Kochel TJ (2009). Usefulness of commercially available GPS data-loggers for tracking human movement and exposure to dengue virus. Int J Health Geogr.

[20] Alarcon V, Alarcon E, Figueroa C, Mendoza-Ticona A (2017). Tubersulosis en el Perú: situación epidemiológica, avances y desafíos para su control. Rev Peru Med Exp Salud Publica.

[21] Grandjean L, Gilman RH, Martin L, Soto E, Castro B, Lopez S (2015). Transmission of multidrug-resistant and drug-susceptible tuberculosis within households: a prospective cohort study. PLoS Med.

[22] Grandjean L, Gilman RH, Iwamoto T, Köser CU, Coronel J, Zimic M (2017). Convergent evolution and topologically disruptive polymorphisms among multidrug-resistant tuberculosis in Peru. PLoS One.

[23] Grandjean L, Monteserin J, Gilman R, Pauschardt J, Bonilla C, Ritacco V, et al. Association between bacterial homoplastic variants and radiological pathology in tuberculosis Tuberculosis. Thorax. 2020. 10.1136/thoraxjnl-2019-213281. 10.1136/thoraxjnl-2019-213281 PMC7361023 32546574

[24] Meehan CJ, Moris P, Kohl TA, Pečerska J, Akter S, Merker M (2018). The relationship between transmission time and clustering methods in Mycobacterium tuberculosis epidemiology. EBioMedicine..

[25] Powell RA, Mitchell MS (2012). What is a home range?. J Mammal.

[26] Fieberg J, Kochanny CO (2005). Quantifying home-range overlap: the importance of the utilization distribution. J Wildl Manag.

[27] Worton BJ (1989). Kernel methods for estimating the utilization distribution in home-range studies. Ecology..

[28] Clapp JG, Beck JL (2015). Evaluating distributional shifts in home range estimates. Ecol Evol.

[29] Walter WD, Onorato DP, Fischer JW (2015). Is there a single best estimator?Selection of home range estimators using area-under-the-curve. Mov Ecol.

[30] Calenge C (2015). Home range estimation in R: the adehabitatHR package.

[31] Bui DP, Oren E, Roe DJ, Brown HE, Harris RB, Knight GM, et al. A Case Control Study to Identify Community Venues Associated with Genetically Clustered Multidrug-Resistant Tuberculosis Disease in Lima, Peru. Clin Infect Dis. 2018:ciy746. 10.1093/cid/ciy746. 10.1093/cid/ciy746 PMC7181380 30239609

[32] Cudahy PGT, Andrews JR, Bilinski A, Dowdy DW, Mathema B, Menzies NA (2019). Spatially targeted screening to reduce tuberculosis transmission in high-incidence settings. Lancet Infect Dis.

[33] Yang C, Lu L, Warren JL, Wu J, Jiang Q, Zuo T (2018). Internal migration and transmission dynamics of tuberculosis in Shanghai, China: an epidemiological, spatial, genomic analysis. Lancet Infect Dis.

[34] Warren JL, Grandjean L, Moore DAJ, Lithgow A, Coronel J, Sheen P (2018). Investigating spillover of multidrug-resistant tuberculosis from a prison: a spatial and molecular epidemiological analysis. BMC Med.

[35] Kestens Y, Thierry B, Shareck M, Steinmetz-Wood M, Chaix B (2018). Integrating activity spaces in health research: comparing the VERITAS activity space questionnaire with 7-day GPS tracking and prompted recall. Spat Spatiotemporal Epidemiol.

[36] Hatherell H-A, Colijn C, Stagg HR, Jackson C, Winter JR, Abubakar I (2016). Interpreting whole genome sequencing for investigating tuberculosis transmission: a systematic review. BMC Med.

[37] Siła-Nowicka K, Vandrol J, Oshan T, Long JA, Demšar U, Fotheringham AS (2016). Analysis of human mobility patterns from GPS trajectories and contextual information. Int J Geogr Inf Sci.

