# Supplementary material for: Community transmission of multidrug-resistant tuberculosis is associated with activity space overlap in Lima, Peru
Source: BMC Infect Dis. 2021 Mar 18;21:275. doi: 10.1186/s12879-021-05953-8 (PMC7977184; doi:10.1186/s12879-021-05953-8)
Supplement: Supplementary file 1 — Additional file 1: Table S1. Distribution of sublineages in study and parent study. Fisher-exact P-value = 0.83. [file 12879_2021_5953_MOESM1_ESM.docx]

| **Table S1.** Distribution of sublineages in study and parent study. Fisher-exact P-value = 0.83. | | | | |
| --- | --- | --- | --- | --- |
|  | **Cases enrolled in current study (N=35)** | | **Cases from parent study population (N=470)** | |
| **Sub Lineage** | **No.** | **%** | **No.** | **%** |
| Beijing | 2 | 5.71 | 34 | 7.2 |
| Haarlem | 4 | 11.4 | 81 | 17.2 |
| LAM | 24 | 68.6 | 255 | 54.3 |
| Other Small Clades | 3 | 8.57 | 47 | 10.0 |
| T | 2 | 5.71 | 47 | 10.0 |
| Bovis | 0 | - | 1 | 0.2 |
| Caprae | 0 | - | 3 | 0.6 |
| EAI2-Manilla | 0 | - | 1 | 0.2 |
| H37Rv | 0 | - | 1 | 0.2 |
