# Supplementary material for: Community transmission of multidrug-resistant tuberculosis is associated with activity space overlap in Lima, Peru
Source: BMC Infect Dis. 2021 Mar 18;21:275. doi: 10.1186/s12879-021-05953-8 (PMC7977184; doi:10.1186/s12879-021-05953-8)
Supplement: Supplementary file 2 — Additional file 2: Table S2. Comparison of mean 95% UDOI by dyad type, excluding clustered cases from the same household. [file 12879_2021_5953_MOESM2_ESM.docx]

| **Table S2.** Comparison of mean 95% UDOI by dyad type, excluding clustered cases from the same household. | | | | | |
| --- | --- | --- | --- | --- | --- |
| **Dyad Type** |  | | | **All Dyads** | **Excluding  Non-overlapping Dyads** |
|  | **N** | **No Overlap, n (%)** | **Overlap, n (%)** | **95% UDOI, mean (sd)** | **95% UDOI, mean (sd)** |
| **Both Regions** | | | | | |
| Both Cases | 595 | 385 (64.7) | 210 (35.3) | 0.06 (0.22) | 0.17 (0.34) |
| Both Controls | 2016 | 1089 (54.0) | 927 (46.0) | 0.14 (0.36) | 0.31 (0.47) |
| Case-Control | 2240 | 1311 (58.5) | 929 (41.5) | 0.06 (0.19) | 0.13 (0.28) |
| Clustered | 14 | 8 (57.14) | 6 (42.86) | 0.19 (0.4) | 0.45 (0.52) |
| Not Clustered | 580 | 377 (65) | 203 (35) | 0.05 (0.2) | 0.15 (0.31) |
| **Callao Only** | | | | | |
| Both Cases | 120 | 68 (56.7) | 52 (43.3) | 0.12 (0.33) | 0.29 (0.45) |
| Both Controls | 496 | 126 (25.4) | 370 (74.6) | 0.29 (0.43) | 0.39 (0.46) |
| Case-Control | 512 | 232 (45.3) | 280 (54.7) | 0.09 (0.23) | 0.16 (0.29) |
| Clustered | 3 | 2 (.67) | 1 (.33) | 0.27 (0.47) | 0.82 (--) |
| Not Clustered | 116 | 66 (56.9) | 50 (43.1) | 0.11 (0.29) | 0.25 (0.4) |
| **Lima Sur Only** | | | | | |
| Both Cases | 171 | 36 (21.1) | 135 (78.9) | 0.12 (0.27) | 0.15 (0.3) |
| Both Controls | 496 | 34 (6.9) | 462 (93.1) | 0.3 (0.49) | 0.32 (0.5) |
| Case-Control | 608 | 79 (13.0) | 529 (87.0) | 0.13 (0.28) | 0.14 (0.3) |
| Clustered | 7 | 2 (28.6) | 5 (71.4) | 0.27 (0.48) | 0.38 (0.55) |
| Not Clustered | 164 | 34 (20.7) | 130 (79.3) | 0.11 (0.26) | 0.14 (0.29) |
