# Supplementary material for: Community transmission of multidrug-resistant tuberculosis is associated with activity space overlap in Lima, Peru
Source: BMC Infect Dis. 2021 Mar 18;21:275. doi: 10.1186/s12879-021-05953-8 (PMC7977184; doi:10.1186/s12879-021-05953-8)
Supplement: Supplementary file 3 — Additional file 3: Table S3. Association between genetic similarity of Mycobacterium tuberculosis strains of two case pairs (measured in number of different single nucleotide polymorphisms (SNP)) and their utilization distribution overlap index (UDOI), excluding clustered cases that are household members. [file 12879_2021_5953_MOESM3_ESM.docx]

| **Table S3.**  Association between genetic similarity of *Mycobacterium tuberculosis* strains of two case pairs (measured in number of different single nucleotide polymorphisms (SNP)) and their utilization distribution overlap index (UDOI), excluding clustered cases that are household members. | | | | |
| --- | --- | --- | --- | --- |
| **Linear regression of Log(SNP) difference on logit(UDOI).** | **Coef.** | **95%CI** | | **P** |
| 50% Home Range UDOI | -0.77 | -1.243 | -0.301 | 0.001 |
| 95% Home Range UDOI | -2.453 | -3.675 | -1.23 | <0.001 |
| 99% Home Range UDOI | -3.459 | -4.898 | -2.02 | <0.001 |
|  | | | | |
| **Odds of being in clustered dyad vs. non-clustered dyad by UDOI.** | **OR** | **95%CI** | | **P** |
| 50% Home Range UDOI | 3.03 | 1.02 | 8.98 | 0.046 |
| 95% Home Range UDOI | 1.17 | 1.02 | 1.34 | 0.023 |
| 99% Home Range UDOI | 1.07 | 1.00 | 1.15 | 0.061 |
